# Synergistic Effect of Dual Particle-Size AuNPs on TiO_2_ for Efficient Photocatalytic Hydrogen Evolution

**DOI:** 10.3390/nano9040499

**Published:** 2019-04-01

**Authors:** Qian Zhao, Qiaoli Zhang, Cui Du, Shasha Sun, Jay D. Steinkruger, Chen Zhou, Shengyang Yang

**Affiliations:** 1 School of Chemistry and Chemical Engineering, Yangzhou University, 180 Siwangting Road, Yangzhou 225002, China; mr.qianzhao@hotmail.com (Q.Z.); zql0823@hotmail.com (Q.Z.); ms.cdu@hotmail.com (C.D.); 2 School of Environmental and Chemical Engineering, Jiangsu University of Science and Technology, Zhenjiang 212018, China; sunshasha@just.edu.cn; 3 School of Natural Sciences, University of Central Missouri, Warrensburg, MO 64093, USA; steinkruger@ucmo.edu

**Keywords:** AuNPs/TiO_2_, synergistic effect, dual-size particle, photocatalytic, hydrogen production

## Abstract

Design of efficient catalysts for photocatalytic water-splitting hydrogen evolution is of fundamental importance for the production of sustainable clean energy. In this study, a dual particle-size AuNPs/TiO_2_ composite containing both small (16.9 ± 5.5 nm) and large (45.0 ± 9.8 nm) AuNPs was synthesized by annealing two different sized AuNPs onto TiO_2_ nanosheets. Dual particle-size AuNPs/TiO_2_ composites of 2.1 wt% catalyze photocatalytic H_2_ evolution 281 times faster than pure TiO_2_. Control experiments indicate the observed rate increase for the 2.1 wt% dual particle-size AuNPs/TiO_2_ composites is larger than 2.1 wt% small AuNPs/TiO_2_ composites, or 2.1 wt% large AuNPs/TiO_2_ composites in isolation. The observed photocatalytic enhancement can be attributed to the synergistic effect of dual particle-size AuNPs on TiO_2_. Specifically, small-sized AuNPs can act as an electron sink to generate more electron-hole pairs, while the surface plasmon resonance (SPR) effect of large-sized AuNPs concurrently injects hot electrons into the TiO_2_ conduction band to enhance charge transfer. In addition, a gold-dicyanodiamine composite (GDC)-directed synthesis of 2.1 wt% dual particle-size AuNPs/TiO_2_ composites was also completed. Notably, a photocatalytic efficiency enhancement was observed that was comparable to the previously prepared 2.1 wt% dual particle-size AuNPs/TiO_2_ composites. Taken together, the synergistic effects of dual particle-size AuNPs on TiO_2_ can be potentially used as a foundation to develop semiconductor photocatalyst heterojunction with enhanced photocatalytic activity.

## 1. Introduction

The development of clean and renewable fuels continues to attract significant attention due to the environmental concern caused by global consumption of fossil fuels. Electrochemical photolysis of water to generate hydrogen (H_2_) using a TiO_2_ electrode was first reported in 1972 [1]. Since that time, photocatalytic H_2_ production has become one of the most promising routes to secure H_2_ as an alternative energy source [2]. Use of TiO_2_ as a photocatalyst was often driven by its low cost and strong oxidizing capacity; however, the high recombination rate of photoinduced electron-hole pairs significantly limited the photocatalytic activity of TiO_2_. Approaches for improving the photocatalytic performance of TiO_2_ have included: (a) heteroatom doping [3]; (b) textural design [4]; and (c) heterojunction formation with metals or other semiconductors [5,6]. Among these approaches, semiconductor heterojunction design, especially co-catalyst coupling, has been extensively investigated [7]. Suitable co-catalysts promote effective transfer of photogenerated electrons by serving as electron reservoirs. Additionally, co-catalysts provide extra active sites for the photocatalytic redox reactions, which results in suppressed electron-hole recombination [8,9].

Gold (Au) has been extensively studied as a co-catalyst of TiO_2_ to enhance photocatalytic performance [10,11,12]. The inclusion of Au allows electrons to be readily trapped and transferred, because its Fermi level is lower than the conduction band of TiO_2_ [13]. Additionally, the surface plasmon resonance (SPR) effect of Au nanoparticles (AuNPs) can broaden the absorption of the photocatalyst [14]. Broader light absorption increases the amount of available “hot electrons” [15], which can bolster photocatalysis performance. Indeed, recent studies have demonstrated that addition of AuNPs as co-catalysts can significantly enhance photocatalytic H_2_ production [16,17]. However, previous studies that utilized conventional deposition–precipitation synthetic methods often focused on investigating single-sized AuNPs as co-catalysts [18,19,20]. Given that SPR effect is strongly dependent on Au particle size [21,22,23], the evaluation of how multi-sized AuNPs co-catalysts on TiO_2_ influence its photocatalytic H_2_ production efficiency is highly desired.

Here, we employed two particle-size populations of AuNPs to decorate TiO_2_ for remarkably enhanced photocatalytic H_2_ evolution. Two kinds of AuNPs with different size populations were first synthesized, which were then utilized to modify pre-prepared TiO_2_ nanosheets via a direct annealing process. After annealing, two scaled AuNPs with the size distributions of 16.9 ± 5.5 nm (“small”) and 45.0 ± 9.8 nm (“large”) were successfully anchored onto TiO_2_. The resulting AuNPs/TiO_2_ photocatalyst (2.1 total wt% AuNPs, 2 wt% large AuNPs + 0.1 wt% small AuNPs) produced a H_2_ evolution efficiency 281 times greater that of TiO_2_ alone. Control experiments indicate that the photocatalytic efficiency of AuNPs/TiO_2_ composites decorated with both small and large AuNPs significantly outperforms AuNPs/TiO_2_ composites containing small or large AuNPs at a total concentration of 2.1 wt% AuNPs on TiO_2_. Inspired by this result, we wondered if analogous H_2_ evolution could be achieved when preparing dual particle-size AuNPs/TiO_2_ photocatalysts using a different synthetic method. We explored an alternative synthetic method for preparing 2.1 wt% AuNPs/TiO_2_ (two similar AuNP particle size distributions) by annealing of gold-dicyanodiamine composites (GDCs) onto TiO_2_. An enhanced photocatalytic efficiency comparable to the above synthesized 2.1 wt% AuNPs/TiO_2_ photocatalyst was observed. Taken together, our results reveal a synergistic effect of the two AuNP particle size distributions when the resulting 2.1 wt% AuNPs/TiO_2_ photocatalysts are used for H_2_ generation. This work may serve as a framework for future semiconductor photocatalyst heterojunction designs for enhanced photocatalytic applications.

## 2. Experimental Section

### 2.1. Materials

Chloroauric acid hydrate (HAuCl_4_·4H_2_O, ≥47.8% Au basis), dicyanodiamine (C_2_H_4_N_4_, ≥98%), sodium borohydride (NaBH_4_, ≥98%), and triethanolamine (C_6_H_15_NO_3_, ≥99.8%) were purchased from Sinopharm Chemical Reagent Co., Ltd. (Shanghai, China) Titanium tetrachloride (TiCl_4_, ≥99.0%) and ethylene glycol (C_2_H_6_O_2_, ≥98.0%) were purchased from Shanghai Macklin Biochemical Co., Ltd. (Shanghai, China) Glycine (C_2_H_5_NO_2_, ≥99.0%) and sodium sulfate (Na_2_SO_4_, ≥99.0%) were purchased from Shanghai Titan Scientific Co., Ltd. (Shanghai, China) All reagents were used without further purification. High-purity water with the resistivity of ≥18.2 MΩ*cm was used in all experiments.

### 2.2. Preparation of TiO_2_ Nanosheets

Titanium tetrachloride (0.125 mmol) was added to a stirring solution of ethylene glycol (EG, 77.5 mL). The solution was stirred until it became clear and no additional HCl gas was generated. Deionized (DI) water (2.5 mL) was added, and the resulting solution was transferred to a 100 mL Teflon-lined autoclave. The reaction mixture was maintained at 150 °C for 4 h. The resulting solid was rinsed with a mixture of DI water and ethanol (volume ratio: 1/2) and subsequently freeze-dried.

### 2.3. Fabrication of Large (45.0 ± 9.8 nm) AuNPs/TiO_2_ Photocatalysts

An aqueous solution (100 mL) containing HAuCl_4_ (12.5 μmol) and glycine (1.25 mmol) was heated at 100 °C for 10 min in a microwave reactor (700 W). The as-prepared AuNPs solution (2.46 mg Au/100 mL H_2_O) was then mixed with TiO_2_ (100 mg) at different Au:TiO_2_ ratios, freeze-dried, and annealed at 550 °C in a tube furnace for 6 h in air with a ramp of 5 °C min^−1^. The wt% values of large sized AuNPs relative to TiO_2_ were 0.1, 0.5, 1.0, 2.0, 2.1, and 3.5 wt%, respectively.

### 2.4. Fabrication of Small (16.9 ± 5.5 nm) AuNPs/TiO_2_ Photocatalysts

A freshly prepared aqueous NaBH_4_ solution (10 mL, 0.5 M) was rapidly added into an aqueous HAuCl_4_ (0.5 mmol, 190 mL) solution under vigorous stirring. The resulting solution was stirred at room temperature (ca. 22 °C) for 2.5 h. The as-obtained AuNPs solution was combined with TiO_2_ (100 mg), vortexed to ensure thorough mixing, and freeze-dried. Annealing was completed at 550 °C in a tube furnace for 6 h in air with a ramp of 5 °C min^−1^. The wt% values of small AuNPs relative to TiO_2_ were 0.02, 0.05, 0.1, 1.0, 2.0, and 2.1 wt% respectively.

### 2.5. Preparation of Dual Particle-Size AuNPs/TiO_2_ Photocatalysts

TiO_2_ (100 mg) was added to a 10 mL aqueous solution containing both 2.0 wt% large AuNPs and 0.1 wt% small AuNPs. The resulting solution was sonicated until thoroughly mixed, freeze-dried, and annealed in a tube furnace at 550 °C for 6 h in air with a ramp of 5 °C min^−1^.

### 2.6. Preparation of GDC-Directed Dual Particle-Size AuNPs/TiO_2_ Photocatalysts

An aqueous solution containing HAuCl_4_ (0.02 M) and dicyanodiamine (0.006 M) was heated at 60 °C for 30 min to synthesize gold-dicyanodiamine composites (GDCs). The resulting solid was rinsed multiple times with DI water. The 2.1 wt% dual particle-size AuNPs/TiO_2_ was also achieved by mixing appropriate amounts of GDCs and TiO_2_ nanosheets, and annealing them in a tube furnace at 550 °C in air for 6 h with a ramp of 5 °C min^−1^.

### 2.7. Characterization Methods

Transmission electron microscopy (TEM) and high-resolution transmission electron microscopy (HRTEM) were performed on a field emission transmission electron microscope (Tecnai G2 F30 S-TWIN, FEI, Hillsboro, OR, USA) at an acceleration voltage of 300 kV. X-ray diffraction (XRD) measurements were completed on an X-ray diffractometer (D8 Advance, BRUKER-AXS, Billerica, MA, USA) with Cu Kα radiation. UV-vis diffuse reflectance spectra (DRS) were obtained with a UV-Vis-NIR spectrophotometer (Cary-5000, Varian, Palo Alto, CA, USA). Photoluminescence (PL) spectra were measured on a spectrofluorometer (F-4500, Hitachi, Tokyo, Japan, Xe lamp as light source). Time-resolved fluorescence spectra were obtained with time-resolved spectroscopy (FLSP20, Edinburgh Instruments, Edinburgh, UK). Surface electronic states and compositions of the samples were analyzed by X-ray photoelectron spectroscopy (XPS, ESCALAB250 Xi, Thermo Scientific, Somerset, NJ, USA). Brunauer-Emmett-Teller (BET) specific surface area (SBET) was determined by nitrogen adsorption-desorption isotherm measurements (ASAP 2020 HD88, Micromeritics, Norcross, GA, USA).

### 2.8. Photocatalytic Activity Evaluation

Photocatalytic activity was evaluated by H_2_ generation from water splitting. The photocatalytic H_2_ evolution reactions were carried out in a Pyrex reactor under vacuum. The temperature of the reactant solution was kept at 6 °C by flowing cooling water. Each as-prepared photocatalyst (10 mg) was dispersed in a mixture of 80 mL DI water and 20 mL triethanolamine solution. Subsequently, the resulting suspensions were illuminated by a 300 W xenon lamp (CEL-HXF 300, CEAULIGHT, Beijing, China, 350–780 nm). Gas chromatography with a thermal conductivity detector (TCD) was employed for H_2_ production analysis using N_2_ as carrier gas.

### 2.9. Electrochemical Characterization

Transient photocurrent and electrochemical impedance spectroscopy (EIS) were conducted in Na_2_SO_4_ (0.1 M) using an electrochemical instrument (Gamry Interface 1010, Warminster, PA, USA) with a traditional three-electrode system under light irradiation (300 W Xe-lamp). The applied photocurrent bias was 0.9 V. The working electrode was prepared by dispersing each photocatalyst (5 mg) in 1 mL of ethanol (containing 25 μL of nafion solution) and dropped on FTO glass. Ag/AgCl and Pt electrodes were used as the reference electrode and counter electrode, respectively.

## 3. Results and Discussion

**Figure 1:**
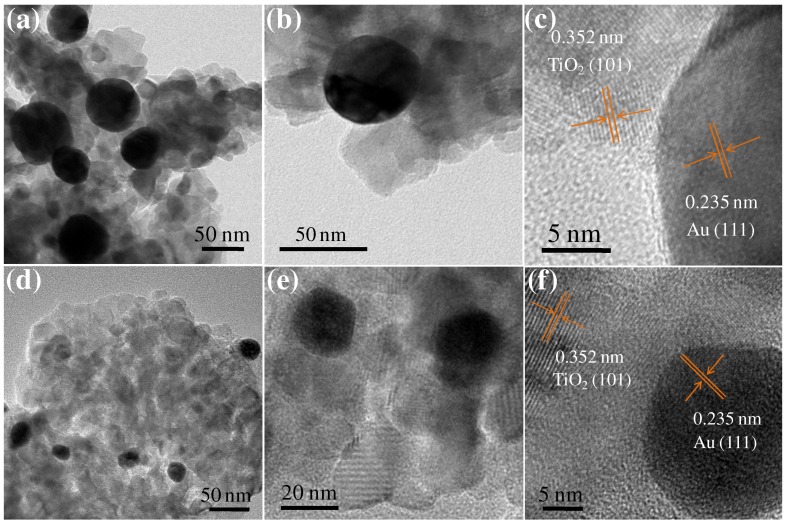
(**a**,**b**) Transmission electron microscopy (TEM) and (**c**) high resolution TEM (HRTEM) photographs of large AuNPs/TiO_2_ hybrids. (**d**,**e**) TEM and (**f**) HRTEM photographs of small AuNPs/TiO_2_ composites.

Large AuNPs/TiO_2_ and small AuNPs/TiO_2_ photocatalysts with different AuNPs weight ratios were synthesized to determine the optimum weight percent of AuNPs for photocatalysis. TEM was utilized to evaluate the microstructures of the prepared photocatalysts. The large AuNPs/TiO_2_ composite (Figure 1a,b) had an average Au nanosphere size of 45.0 ± 9.8 nm (Figure S1), while the small AuNPs/TiO_2_ composite (Figure 1d,e) had an average Au nanosphere size of 16.9 ± 5.5 nm (Figure S2). Both composites appeared to be well distributed onto the TiO_2_ matrix. The 0.235 nm lattice spacing observed in these composites can be ascribed to the d spacing of Au (111) crystal plane, while the 0.352 nm lattice spacing belongs to the TiO_2_ (101) crystal plane (Figure 1c,f) [24,25].

**Figure 2:**
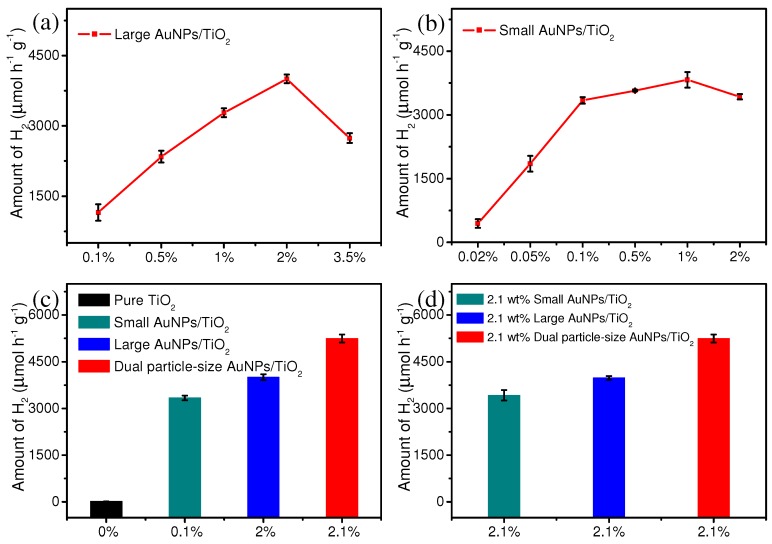
(**a**,**b**) Photocatalytic H_2_ generation rates for different concentrations of large AuNPs/TiO_2_ and small AuNPs/TiO_2_ photocatalysts under light irradiation (350–780 nm). (**c**) Photocatalytic H_2_ generation rates of pure TiO_2_, small AuNPs/TiO_2_, large AuNPs/TiO_2_, and dual particle-size AuNPs/TiO_2_ photocatalysts under light irradiation (350–780 nm). (**d**) Photocatalytic H_2_ generation rates of 2.1 wt% small AuNPs/TiO_2_, 2.1 wt% large AuNPs/TiO_2_, and 2.1 wt% dual particle-size AuNPs/TiO_2_ under light irradiation (350–780 nm).

Photocatalytic performance of all synthesized photocatalysts was evaluated by measuring H_2_ evolution rates from water splitting. Each photocatalyst was evaluated for 3 h to minimize experimental errors. The H_2_ evolution rate using large AuNPs/TiO_2_ composites increased up to 2 wt% AuNPs and then decreased at 3.5 wt% (Figure 2a). The highest H_2_ evolution rate of 4006 µmol h^−1^ g^−1^ (2 wt% AuNPs) was approximately 215 times greater than that of TiO_2_ (18.67 µmol h^−1^ g^−1^) under the same conditions. Small AuNPs/TiO_2_ composites exhibited increasing H_2_ evolution up to 1 wt% AuNPs; however, no significant increase was observed above 0.1 wt% (Figure 2b). The 0.1 wt% small AuNPs/TiO_2_ composites exhibited H_2_ production with a generation rate of 3340 µmol h^−1^ g^−1^, which was approximately 179 times greater than that of pure TiO_2_.

Based on the observations above, we hypothesized that a dual particle-size AuNPs/TiO_2_ composite (2 wt% large AuNPs + 0.1 wt% small AuNPs) could exhibit a synergistic effect that would further increase the rate of H_2_ evolution. Indeed, a hydrogen evolution rate of 5245 µmol h^−1^ g^−1^ was higher than the rate observed with either single particle-size AuNPs/TiO_2_ composite (Figure 2c). Control experiments with 2.1 wt% small AuNPs/TiO_2_ or 2.1 wt% large AuNPs/TiO_2_ failed to achieve the H_2_ evolution rate observed for the 2.1 wt% dual particle-size AuNPs/TiO_2_ composite (Figure 2d). These results indicate the fabrication of both small and large AuNPs populations onto TiO_2_ appears to lead to a synergistic effect for photocatalytic H_2_ evolution.

**Figure 3:**
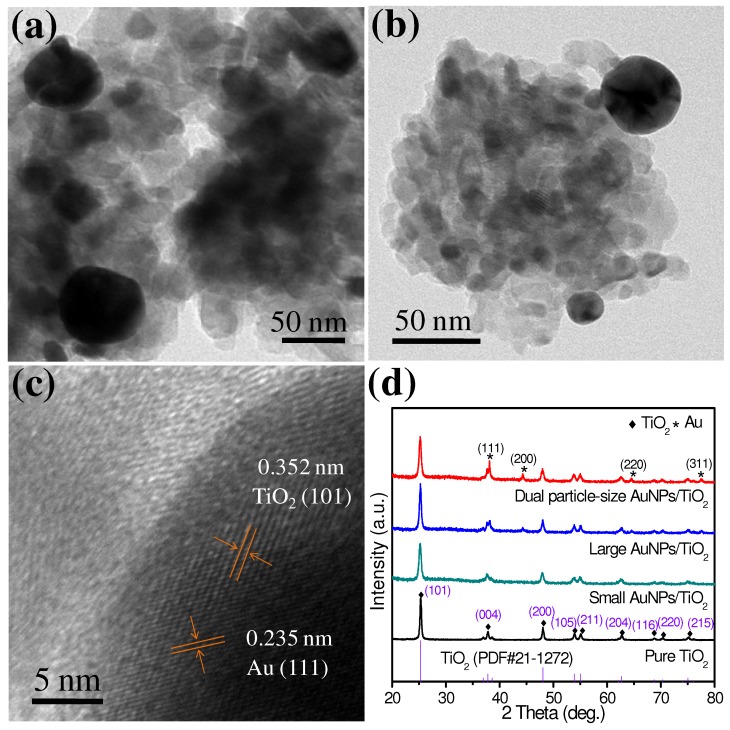
(**a**,**b**) TEM and (**c**) HRTEM photographs of 2.1 wt% dual particle-size AuNPs/TiO_2_ hybrids. (**d**) X-ray diffraction (XRD) patterns of pure TiO_2_, small AuNPs/TiO_2_, large AuNPs/TiO_2_, and dual particle-size AuNPs/TiO_2_ hybrids.

The successful fabrication of a dual particle-size 2.1 wt% AuNPs/TiO_2_ composite was confirmed by TEM and HRTEM images (Figure 3). Two distinguishable AuNP size distributions, of 17.3 ± 7.5 nm and 46.3 ± 6.1 nm, were found by measuring 100 individual AuNPs (Figure S3). Significantly, these particle-size distributions were very similar to those observed with the single particle-size composites described above. The Au (111) (0.235 nm) and TiO_2_ (101) (0.352 nm) crystal planes seen in Figure 3c indicated that both Au and TiO_2_ were successfully incorporated in the composite structure. The crystal phase of pure TiO_2_, 0.1 wt% small AuNPs/TiO_2_, 2 wt% large AuNPs/TiO_2_, and 2.1 wt% dual particle-size AuNPs/TiO_2_ were investigated using powder XRD. All XRD patterns showed several characteristic peaks at 25.3°, 37.8°, 48.0°, 53.9°, 55.1°, 62.7°, 68.8°, 70.3°, and 75.0°, corresponding to the (101), (004), (200), (105), (211), (204), (116), (220), and (215) planes of the anatase phase of TiO_2_ (Figure 3d). The large AuNPs/TiO_2_ and dual-particle size AuNPs/TiO_2_ also displayed peaks at 38.2°, 44.4°, 64.6°, and 77.5°, which can be indexed to the (111), (200), (220), and (311) planes of Au. These observations were consistent with TEM results and provide additional evidence of successful fabrication of AuNPs onto TiO_2_ [26,27]. Small AuNPs/TiO_2_; however, exhibited no significant difference from TiO_2_. We hypothesize this is due to the low content (0.1 wt%) of small AuNPs on TiO_2_. 

**Figure 4:**
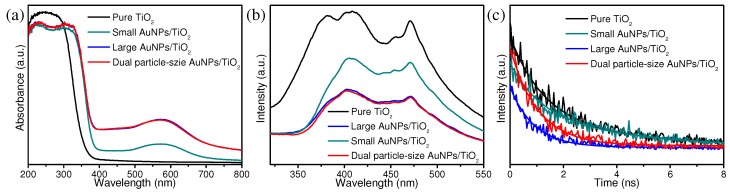
(**a**) UV-vis diffuse-reflectance spectra, (**b**) photoluminescence spectra, and (**c**) time-resolved luminescence decay spectra of pure TiO_2_, small AuNPs/TiO_2_, large AuNPs/TiO_2_, and dual particle-size AuNPs/TiO_2_ hybrids.

UV-vis diffuse reflectance spectra (DRS) and Photoluminescence (PL) spectra were collected to gain insight into the relationship between optical and photocatalytic properties of the synthesized materials. As shown in Figure 4a, the photocatalysts containing AuNPs exhibited a light absorption range significantly beyond the 390 nm absorption edge of pure TiO_2_ [28,29,30,31]. In addition, a typical absorption peak around 580 nm can be observed from all three AuNPs/TiO_2_ hybrids, corresponding to the SPR of AuNPs [32]. PL spectra displayed fluorescence quenching from all three AuNPs/TiO_2_ hybrids (Figure 4b). These results suggest the deposition of AuNPs onto TiO_2_ can efficiently restrain the electron-hole recombination [33,34], which leads to faster transfer of electrons between the TiO_2_ and the AuNPs. In addition, the time-resolved fluorescence decay spectra of all samples were also obtained (Figure 4c). The results suggest all four photocatalysts possessed similar fluorescence lifetimes; however, changes to the band gap of TiO_2_ were observed after introducing different-sized AuNPs (Figure S4), indicating coupling AuNPs onto TiO_2_ could increase the utilization of light [35]. The large AuNPs/TiO_2_ and dual particle-size AuNPs/TiO_2_ exhibited higher absorption intensity than that of small AuNPs/TiO_2_. This observation can be attributed to the stronger electron-trapping effect caused by the SPR of large AuNPs populations. Additionally, obvious reduction in PL emission intensity was observed from both the large and dual particle-size AuNPs/TiO_2_, likely resulting from the stronger electron-hole separation effect in these two photocatalysts. Both observations are consistent with the photocatalytic testing results, in which the large AuNPs/TiO_2_ and dual particle-size AuNPs/TiO_2_ composites exhibit higher photocatalytic efficiency than that of the small AuNPs/TiO_2_ composites. However, no major optical property difference was observed between large AuNPs/TiO_2_ and dual particle-sized AuNPs/TiO_2_. As such, the improved photocatalytic properties of the dual particle-size AuNPs/TiO_2_ must emerge from synergistic interactions between the two AuNPs populations on the TiO_2_ surface.

**Figure 5:**
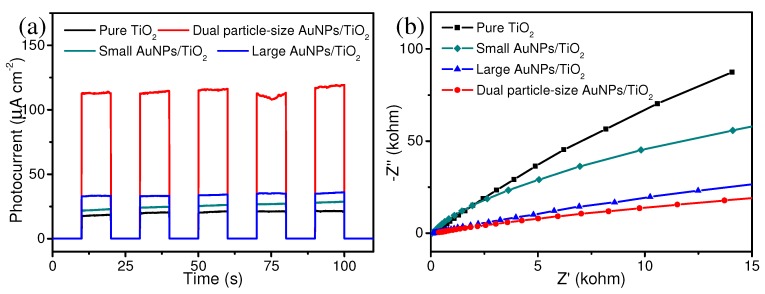
(**a**) Transient photocurrent responses and (**b**) electrochemical impedance spectroscopy Nyquist plots of pure TiO_2_, 0.1 wt% small AuNPs/TiO_2_, 2 wt% large AuNPs/TiO_2_, and 2.1 wt% dual particle-size AuNPs/TiO_2_ photocatalysts under light irradiation (350–780 nm).

Electrochemical measurements of photocurrent responses were collected for all four samples to further probe the enhanced photocatalytic activity of the dual particle-size AuNPs/TiO_2_ composite (Figure 5a). Both 2 wt% large AuNPs/TiO_2_ and 0.1 wt% small AuNPs/TiO_2_ composites demonstrated enhanced photocurrent density than that of pure TiO_2_, implying a higher separation efficiency of photogenerated charge carriers from large AuNPs/TiO_2_ and small AuNPs/TiO_2_ [36]. Significantly, the photocurrent density observed from 2.1 wt% dual particle-size AuNPs/TiO_2_ was almost three times greater than the photocurrent observed from the 2 wt% large AuNPs/TiO_2_ sample. This result strongly suggests that the co-existence of dual particle-size AuNPs on TiO_2_ streamlines charge separation and photogenerated electron transfer. EIS measurements were also carried out under light irradiation (350–780 nm) to provide additional evidence in support of this observation. EIS Nyquist plots of pure TiO_2_, 0.1 wt% small AuNPs/TiO_2_, 2 wt% large AuNPs/TiO_2_, and 2.1 wt% dual particle-size AuNPs/TiO_2_ displayed gradually decreased semicircle radius (Figure 5b). Since the resistance of charge transfer was directly proportional to the semicircle radius of the Nyquist plot [36,37], the introduction of 2.1 wt% dual particle-size AuNPs onto TiO_2_ could enhance the charge transfer separation and inhibit the recombination of photogenerated electron-hole pairs. Taken together, it appears the population of small AuNPs dispersed on TiO_2_ can readily capture electrons generated from the conduction band of TiO_2_; this phenomenon leads to the generation of more electron-hole pairs (narrower band gap) and much easier electron migration [16,38]. Secondly, the large AuNPs offer intensive local electric fields through SPR [39]. Hot electrons formed can be injected into the conduction band of TiO_2_, which greatly facilitates electron transfer and light use efficiency.

**Figure 6:**
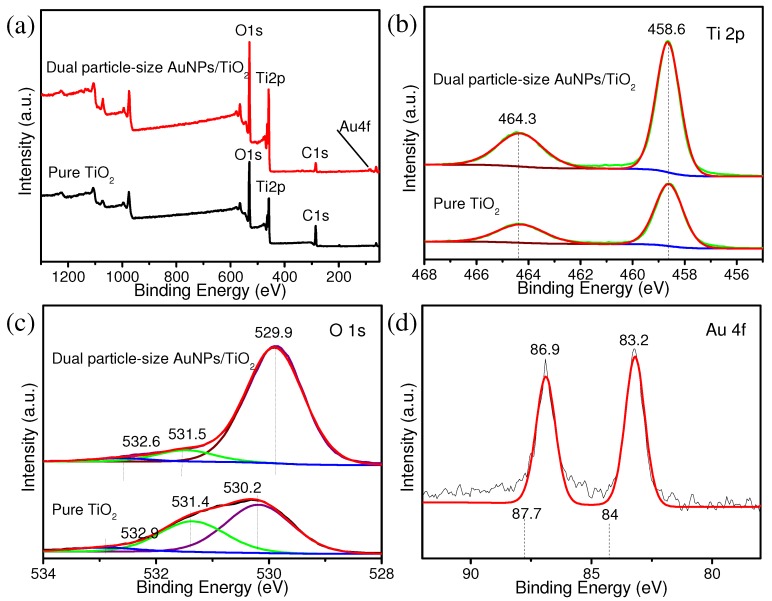
(**a**) Survey X-ray photoelectron spectroscopy (XPS) spectrum and high-resolution spectra of (**b**) Ti 2p, (**c**) O 1s, and (**d**) Au 4f for pure TiO_2_ and dual particle-size AuNPs/TiO_2_ photocatalysts.

Figure S5 shows the N_2_ adsorption-desorption isotherms of all four samples. All isotherms featured typical type-IV curves; however, the pure TiO_2_ sample was found to have a specific surface area of 17.09 m^2^ g^−1^. The small AuNPs/TiO_2_, large AuNPs/TiO_2_, and dual particle-size AuNPs/TiO_2_ exhibited BET surface areas of 58.82 m^2^ g^−1^, 43.23 m^2^ g^−1^, and 11.77 m^2^ g^−1^, respectively. Surface area results suggest the photocatalytic activity enhancement of dual particle-sized AuNPs/TiO_2_ did not originate from a change in surface area [40]. XPS measurements were also employed to analyze the surface electron states in the dual particle-size AuNPs/TiO_2_ composites. The survey XPS spectrum of the dual particle-size AuNPs/TiO_2_ and pure TiO_2_ is shown in Figure 6a. The weak Au signal was mainly due to the low content of AuNPs on the TiO_2_ surface. The peak position of Ti 2p (458.6 and 464.3 eV) in AuNPs/TiO_2_ composites shows no shift compared to pure TiO_2_ (Figure 6b). A 0.3 eV negative shift in the O 1s peaks at 532.9 and 530.2 eV (Figure 6c), as well as a 0.8 eV negative shift from standard Au 4f^0^_5/2_ and Au 4f^0^_7/2_ (Figure 6d), were observed in the dual particle-size AuNPs/TiO_2_ composites. These perturbations were consistent with an increase in electron density for Au and O^2−^. XPS results can be taken as direct evidence for the formation of a Schottky barrier between the AuNPs and TiO_2_ [36,41,42].

**Figure 7:**
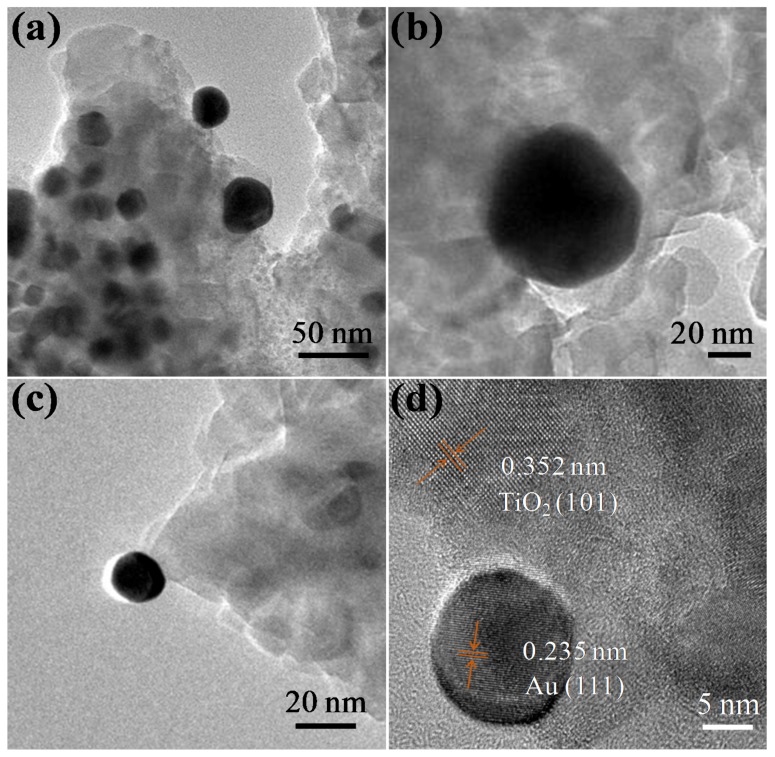
(**a**–**c**) TEM and (**d**) HRTEM photographs of 2.1 wt% gold-dicyanodiamine composite (GDC)-directed dual particle-size AuNPs/TiO_2_ composite.

**Figure 8:**
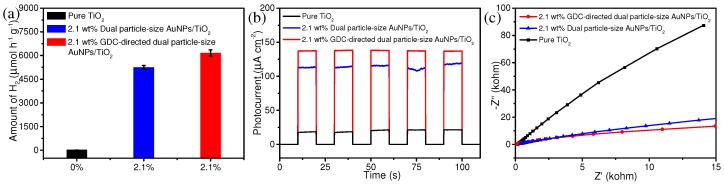
(**a**) Photocatalytic H_2_ generation rates, (**b**) transient photocurrent responses, and (**c**) electrochemical impedance spectroscopy Nyquist plots of pure TiO_2_, 2.1 wt% dual particle-size AuNPs/TiO_2_, and 2.1 wt% GDC-directed dual particle-size AuNPs/TiO_2_ photocatalysts under light irradiation (350–780 nm).

Based on the results described above, we wondered if analogous H_2_ evolution rates could be observed for dual-particle sized AuNPs/TiO_2_ composites prepared by a different synthetic method. Such a result would provide evidence that dual-particle fabrication may be beneficial for AuNPs/TiO_2_ composites regardless of the chosen synthetic strategy. With this in mind, we prepared a 2.1 wt% dual-sized AuNPs/TiO_2_ composite by introducing GDCs onto the TiO_2_ matrix. TEM images of the as-prepared GDCs revealed a uniform spherical structure (diameter ~450 nm) with homogenous incorporation of 3–5 nm AuNPs (Figure S6a). After annealing GDCs to TiO_2_, both large-sized AuNPs and small-sized AuNPs were observed on the TiO_2_ surface (Figure 7). AuNP size distributions were determined to be 45.7 ± 2.9 nm and 16.3 ± 2.8 nm by measuring 100 individual AuNPs (Figure S7). Notably, these two size distributions were very similar to the size distributions of AuNPs on the previously synthesized dual particle-size AuNPs/TiO_2_ composites. XRD experiments (Figure S8) revealed characteristic Au peaks of (111), (200), (220), and (311) crystal planes, which confirms the successful incorporation of AuNPs onto the TiO_2_ matrix. Photocatalytic H_2_ experiments using GDC-directed dual particle-size AuNPs/TiO_2_ photocatalysts revealed a H_2_ evolution rate slightly higher (6150 µmol h^−1^ g^−1^) than that observed from the previously prepared dual particle-size AuNPs/TiO_2_ photocatalysts (Figure 8a). All additional characterization experiments indicated both dual particle-size AuNPs/TiO_2_ composites have analogous electrochemical properties (Figure 8b,c). BET surface area measurements of the 2.1 wt% GDC-directed AuNPs/TiO_2_ composite indicated no alternation of specific surface area after AuNPs incorporation, similar to that of 2.1 wt% dual particle-size AuNPs/TiO_2_ (Figure S9). Binding energy shifts and intensity changes observed from XPS spectra (Figure S10) showed strong interactions between AuNPs and TiO_2_ in the 2.1 wt% GDC-directed AuNPs/TiO_2_ composite for photocatalytic enhancement, consistent with the result obtained from the 2.1 wt% dual particle-size AuNPs/TiO_2_ photocatalysts.

**Figure 9:**
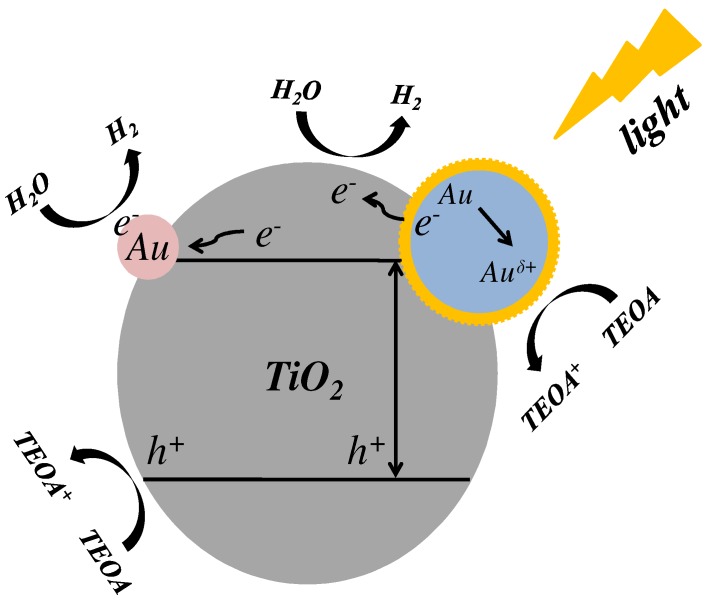
Schematic diagram for possible mechanism of dual particle-size AuNPs/TiO_2_ composites for H_2_ evolution.

A feasible mechanism for the enhanced H_2_ evolution performance from both dual particle-size AuNPs/TiO_2_ composites is presented in Figure 9. Initially, photo-excited electron-hole pairs are generated from the conduction and valence band of TiO_2_ under light irradiation. The generated electrons are then readily captured and transferred to AuNPs [34,43], which contributes to the formation of a Schottky barrier between AuNPs and TiO_2_. Specifically, small-sized AuNPs dispersed on TiO_2_ can promote the capture of electrons generated in the TiO_2_ conduction band, which forms more electron-hole pairs and facilitate the charge transfer [44,45]. Concurrently, an intensive local electric field near large-sized AuNPs can be formed though SPR [15]. Consequently, the hot electrons can be injected into the conduction band of TiO_2_ and significantly boost the charge transfer [19]. As a result, the captured electrons in small sized AuNPs, as well as the transferred electrons from large sized AuNPs to TiO_2_, can both synergistically promote the reduction of H_2_O to H_2_ (holes are consumed by sacrificial agent TEOA).

## 4. Conclusions

In summary, we successfully synthesized a 2.1 wt% dual particle-size AuNPs/TiO_2_ composite and investigated the synergistic effects of the two different AuNPs size distributions on photocatalytic H_2_ production enhancement. The 2.1 wt% dual particle-size AuNPs/TiO_2_ composites not only display a H_2_ evolution efficiency 281 times greater than TiO_2_ alone, but also outperform the AuNPs/TiO_2_ composites containing small or large AuNPs at a total concentration of 2.1 wt% AuNPs. These synergistic effects appear to be attributed to increased electron-hole pairs promoted by small sized AuNPs, as well as the enhanced electron transfer facilitated through the SPR of large AuNPs. Significantly, a comparable photocatalytic efficiency enhancement can be also observed from dual particle-size AuNPs/TiO_2_ photocatalysts synthesized through a different GDC-directed synthetic method. The synergistic effects observed here from dual particle-size AuNPs composites may open a new pathway for designing other efficient photocatalysts in the future.

## Supplementary Materials

The following are available online at https://www.mdpi.com/2079-4991/9/4/499/s1, Figure S1: (a–c) TEM images of large AuNPs/TiO_2_ and (d) Diameter distribution of large sized AuNPs determined by measuring 100 individual AuNPs on TiO_2_. Figure S2: (a) TEM image of small AuNPs/TiO_2_ and (b) Diameter distribution of small sized AuNPs determined by measuring 100 individual AuNPs on TiO_2_. Figure S3: (a–c) TEM images of dual particle-size AuNPs/TiO_2_ and (d) Diameter distribution of dual particle-size AuNPs determined by measuring 100 individual AuNPs on TiO_2_. Figure S4: Transformed Kubelka-Munk function vs. the light energy curves of (a) pure TiO_2_, (b) small AuNPs/TiO_2_, (c) large AuNPs/TiO_2_ and (d) dual particle-size AuNPs/TiO_2_. Figure S5: Nitrogen adsorption of pure TiO_2_, large AuNPs/TiO_2_, small AuNPs/TiO_2_ and dual particle-size AuNPs/TiO_2_. Figure S6: (a) TEM and (b,c) HRTEM images of gold-dicyanodiamine composites (GDCs). Figure S7: (a–c) TEM images of 2.1 wt% GDC-directed dual particle-size AuNPs/TiO_2_ and (d) Diameter distribution of 2.1 wt% GDC-directed dual particle-size AuNPs determined by measuring 100 individual AuNPs on TiO_2_. Figure S8: XRD patterns of pure TiO_2_ and 2.1 wt% GDC-directed dual particle-size AuNPs/TiO_2_. Figure S9: Nitrogen adsorption of 2.1 wt% dual particle-size AuNPs/TiO_2_ and 2.1 wt% GDC-directed dual particle-size AuNPs/TiO_2_. Figure S10: (a) Survey XPS spectrum and high-resolution spectra of (b) Ti 2p, (c) O 1s and (d) Au 4f for pure TiO_2_ and 2.1 wt% GDC-directed dual particle-size AuNPs/TiO_2_.

## References

[1] Fujishima A., Honda K. (1972). Electrochemical photolysis of water at a semiconductor electrode. Nature.

[2] Lu Q., Yu Y., Ma Q., Chen B., Zhang H. (2015). 2D transition-metal-dichalcogenide-nanosheet-based composites for photocatalytic and electrocatalytic hydrogen evolution reactions. Adv. Mater..

[3] Cui G., Wang W., Ma M., Xie J., Shi X., Deng N., Xin J., Tang B. (2015). IR-driven photocatalytic water splitting with WO_2_-Na_x_WO_3_ hybrid conductor material. Nano Lett..

[4] Yu J., Ma T., Liu S. (2011). Enhanced photocatalytic activity of mesoporous TiO_2_ aggregates by embedding carbon nanotubes as electron-transfer channel. Phys. Chem. Chem. Phys..

[5] Irfan R.M., Jiang D., Sun Z., Zhang L., Cui S., Du P. (2017). Incorporating a molecular co-catalyst with a heterogeneous semiconductor heterojunction photocatalyst: Novel mechanism with two electron-transfer pathways for enhanced solar hydrogen production. J. Catal..

[6] Cerro-Prada E., García-Salgado S., Quijano M.Á., Varela F. (2019). Controlled synthesis and microstructural properties of sol-gel TiO_2_ nanoparticles for photocatalytic cement composites. Nanomaterials.

[7] Wang H., Zhang L., Chen Z., Hu J., Li S., Wang Z., Liu J., Wang X. (2014). Semiconductor heterojunction photocatalysts: Design, construction, and photocatalytic performances. Chem. Soc. Rev..

[8] Osterloh F.E. (2013). Inorganic nanostructures for photoelectrochemical and photocatalytic water splitting. Chem. Soc. Rev..

[9] Yang J., Wang D., Han H., Li C. (2013). Roles of cocatalysts in photocatalysis and photoelectrocatalysis. Acc. Chem. Res..

[10] Zheng Z., Huang B., Qin X., Zhang X., Dai Y., Whangboc M.H. (2011). Facile in situ synthesis of visible-light plasmonic photocatalysts M@TiO_2_ (M = Au, Pt, Ag) and evaluation of their photocatalytic oxidation of benzene to phenol. J. Mater. Chem..

[11] Kamat P.V. (2002). Photophysical, photochemical and photocatalytic aspects of metal nanoparticles. J. Phys. Chem. B.

[12] Iwase A., Kato H., Kudo A. (2013). The effect of Au cocatalyst loaded on La-doped NaTaO_3_ on photocatalytic water splitting and O_2_ photoreduction. Appl. Catal. B Environ..

[13] Subramanian V., Wolf E.E., Kamat P.V. (2004). Catalysis with TiO_2_/gold nanocomposites. Effect of metal particle size on the Fermi level equilibration. J. Am. Chem. Soc..

[14] Radzig M., Koksharova O., Khmel I., Ivanov V., Yorov K., Kiwi J., Rtimi S., Tastekova E., Aybush A., Nadtochenko V. (2019). Femtosecond spectroscopy of Au hot-electron injection into TiO_2_: Evidence for Au/TiO_2_ plasmon photocatalysis by bactericidal Au ions and related phenomena. Nanomaterials.

[15] Clavero C. (2014). Plasmon-induced hot-electron generation at nanoparticle/metal-oxide interfaces for photovoltaic and photocatalytic devices. Nat. Photonics.

[16] Liu L., Ouyang S., Ye J. (2013). Gold-nanorod-photosensitized titanium dioxide with wide-range visible-light harvesting based on localized surface plasmon resonance. Angew. Chem. Int. Ed..

[17] Costi R., Saunders A.E., Elmalem E., Salant A., Banin U. (2008). Visible light-induced charge retention and photocatalysis with hybrid CdSe-Au nanodumbbells. Nano Lett..

[18] Murdoch M., Waterhouse G.I.N., Nadeem M.A., Metson J.B., Keane M.A., Howe R.F., Llorca J., Idriss H. (2011). The effect of gold loading and particle size on photocatalytic hydrogen production from ethanol over Au/TiO_2_ nanoparticles. Nat. Chem..

[19] Zhang Q., Yang S., Yin S., Xue H. (2017). Over two-orders of magnitude enhancement of the photocatalytic hydrogen evolution activity of carbon nitride via mediator-free decoration with gold-organic microspheres. Chem. Commun..

[20] Marchal C., Cottineau T., Méndez-Medrano M.G., Colbeau-Justin C., Caps V., Keller V. (2018). Au/TiO_2_-gC_3_N_4_ nanocomposites for enhanced photocatalytic H_2_ production from water under visible light irradiation with very low quantities of sacrificial agents. Adv. Energy Mater..

[21] Kelly K.L., Coronado E., Zhao L., Schatz G.C. (2003). The optical properties of metal nanoparticles: The influence of size, shape, and dielectric environment. J. Phys. Chem. B.

[22] Silva C.G., Juárez R., Marino T., Molinari R., García H. (2010). Influence of excitation wavelength (UV or visible light) on the photocatalytic activity of titania containing gold nanoparticles for the generation of hydrogen or oxygen from water. J. Am. Chem. Soc..

[23] Sun L., Lv P., Li H., Wang F., Su W., Zhang L. (2018). One-step synthesis of Au-Ag alloy nanoparticles using soluble starch and their photocatalytic performance for 4-nitrophenol degradation. J. Mater. Sci..

[24] Wang H., You T., Shi W., Li J., Guo L. (2012). Au/TiO_2_/Au as a plasmonic coupling photocatalyst. J. Phys. Chem. C.

[25] Yang S., Zhou C., Liu J., Yu M., Zheng J. (2012). One-Step interfacial synthesis and assembly of ultrathin luminescent AuNPs/Silica membranes. Adv. Mater..

[26] Sun X., Dong S., Wang E. (2004). Large-scale synthesis of micrometer-scale single-crystalline Au plates of nanometer thickness by a wet-chemical route. Angew. Chem. Int. Ed..

[27] Zhuang Z., Sheng W., Yan Y. (2014). Synthesis of monodispere Au@Co_3_O_4_ core-shell nanocrystals and their enhanced catalytic activity for oxygen evolution reaction. Adv. Mater..

[28] Manga K.K., Zhou Y., Yan Y., Loh K.P. (2009). Multilayer hybrid films consisting of alternating graphene and titania nanosheets with ultrafast electron transfer and photoconversion properties. Adv. Funct. Mater..

[29] Oros-Ruiz S., Zanella R., López R., Hernández-Gordillo A., Gómez R. (2013). Photocatalytic hydrogen production by water/methanol decomposition using Au/TiO_2_ prepared by deposition-precipitation with urea. J. Hazard. Mater..

[30] Bumajdad A., Madkour M., Abdel-Moneam Y., El-Kemary M. (2014). Nanostructured mesoporous Au/TiO_2_ for photocatalytic degradation of a textile dye: The effect of size similarity of the deposited Au with that of TiO_2_ pores. J. Mater. Sci..

[31] Chen R., Lu J., Liu S., Zheng M., Wang Z. (2018). The preparation of Cu_2_O@Au yolk/shell structures for efficient photocatalytic activity with a self-generated acid etching method. J. Mater. Sci..

[32] Torimoto T., Horibe H., Kameyama T., Okazaki K., Ikeda S., Matsumura M., Ishikawa A., Ishihara H. (2011). Plasmon-enhanced photocatalytic activity of cadmium sulfide nanoparticle immobilized on silica-coated gold particles. J. Phys. Chem. Lett..

[33] Chen W., Chang H., Lu J., Huang Y., Harroun S.G., Tseng Y., Li Y., Huang C., Chang H. (2015). Self-assembly of antimicrobial peptides on gold nanodots: Against multidrug-resistant bacteria and wound-healing application. Adv. Funct. Mater..

[34] Kang Y., Yang Y., Yin L., Kang X., Liu G., Cheng H. (2015). An amorphous carbon nitride photocatalyst with greatly extended visible-light-responsive range for photocatalytic hydrogen generation. Adv. Mater..

[35] Jovic V., Chen W., Sun-Waterhouse D., Blackford M.G., Idriss H., Waterhouse G.I.N. (2013). Effect of gold loading and TiO_2_ support composition on the activity of Au/TiO_2_ photocatalysts for H_2_ production from ethanol-water mixtures. J. Catal..

[36] Xu Q., Zeng J., Wang H., Lia X., Xu J., Wu J., Xiao G., Xiao F., Liu X. (2016). Ligand-triggered electrostatic self-assembly of CdS nanosheet/Au nanocrystal nanocomposites for versatile photocatalytic redox applications. Nanoscale.

[37] Xiao F., Miao J., Liu B. (2014). Layer-by-layer self-assembly of CdS quantum dots/graphene nanosheets hybrid films for photoelectrochemical and photocatalytic applications. J. Am. Chem. Soc..

[38] Qu L., Wang N., Xu H., Wang W., Liu Y., Kuo L., Yadav T.P., Wu J., Joyner J., Song Y. (2017). Gold nanoparticles and g-C_3_N_4_-intercalated graphene oxide membrane for recyclable surface enhanced raman scattering. Adv. Funct. Mater..

[39] Rather R.A., Singh S., Pal B. (2017). Visible and direct sunlight induced H_2_ production from water by plasmonic Ag-TiO_2_ nanorods hybrid interface. Sol. Energy Mater. Sol. C.

[40] Tamiolakis I., Fountoulaki S., Vordos N., Lykakisb I.N., Armatas G.S. (2013). Mesoporous Au-TiO_2_ nanoparticle assemblies as efficient catalysts for the chemoselective reduction of nitro compounds. J. Mater. Chem. A.

[41] Kruse N., Chenakin S. (2011). XPS characterization of Au/TiO_2_ catalysts: Binding energy assessment and irradiation effects. Appl. Catal. A Gen..

[42] Rogers C., Perkins W.S., Veber G., Williams T.E., Cloke R.R., Fischer F.R. (2017). Synergistic enhancement of electrocatalytic CO_2_ reduction with gold nanoparticles embedded in functional graphene nanoribbon composite electrodes. J. Am. Chem. Soc..

[43] Yu G., Wang X., Cao J., Wu S., Yan W., Liu G. (2016). Plasmonic Au nanoparticles embedding enhances the activity and stability of CdS for photocatalytic hydrogen evolution. Chem. Commun..

[44] Bhardwaj S., Pal A., Chatterjee K., Rana T., Bhattacharya G., Roy S., Chowdhury P., Sharma G., Biswas S. (2018). Fabrication of efficient dye-sensitized solar cells with photoanode containing TiO_2_-Au and TiO_2_-Ag plasmonic nanocomposites. J. Mater. Sci..

[45] Kumar D.P., Reddy N.L., Karthik M., Neppolian B., Madhavan J., Shankar M.V. (2016). Solar light sensitized p-Ag_2_O/n-TiO_2_ nanotubes heterojunction photocatalysts for enhanced hydrogen production in aqueous-glycerol solution. Sol. Energy Mater. Sol. C.

