# Supplementary material for: Synergistic Effect of Dual Particle-Size AuNPs on TiO2 for Efficient Photocatalytic Hydrogen Evolution
Source: Nanomaterials (Basel). 2019 Apr 1;9(4):499. doi: 10.3390/nano9040499 (PMC6523663; doi:10.3390/nano9040499)
Supplement: Supplementary file 1 [file nanomaterials-09-00499-s001.pdf]

Electronic Supplementary Information

Synergistic Effect of Dual Particle-Size AuNPs on TiO_2 for Efficient Photocatalytic Hydrogen Evolution

Qian Zhao,[1] Qiaoli Zhang,[1] Cui Du,[1] Shasha Sun,[3] Jay D. Steinkruger,[2] Chen Zhou,[1,2,*] and Shengyang Yang[1,*]

[1]School of Chemistry and Chemical Engineering, Yangzhou University, 180 Siwangting Road, Yangzhou, Jiangsu, 225002, P. R. China.

[2]School of Natural Sciences, University of Central Missouri, Warrensburg, Missouri 64093, USA.

[3]School of Environmental and Chemical Engineering, Jiangsu University of Science and Technology, Zhenjiang, 212018, PR China.

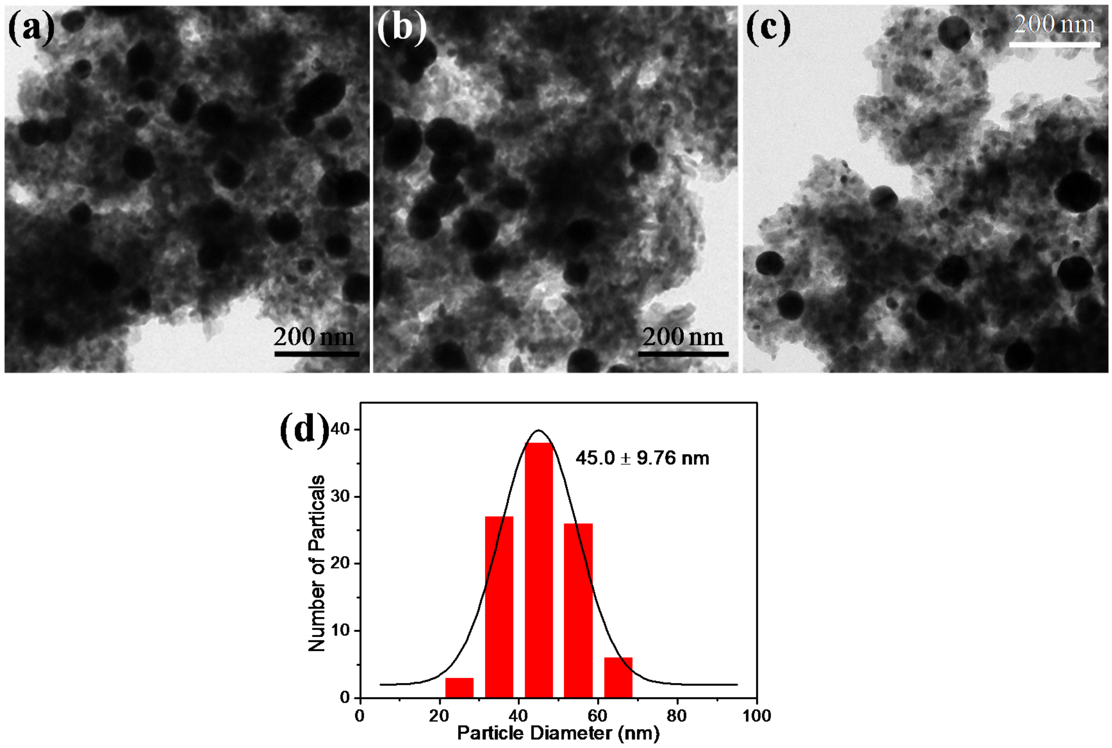

Fig. S1 (a-c) TEM images of large AuNPs/TiO_2 and (d) Diameter distribution of large sized AuNPs determined by measuring 100 individual AuNPs on TiO_2.

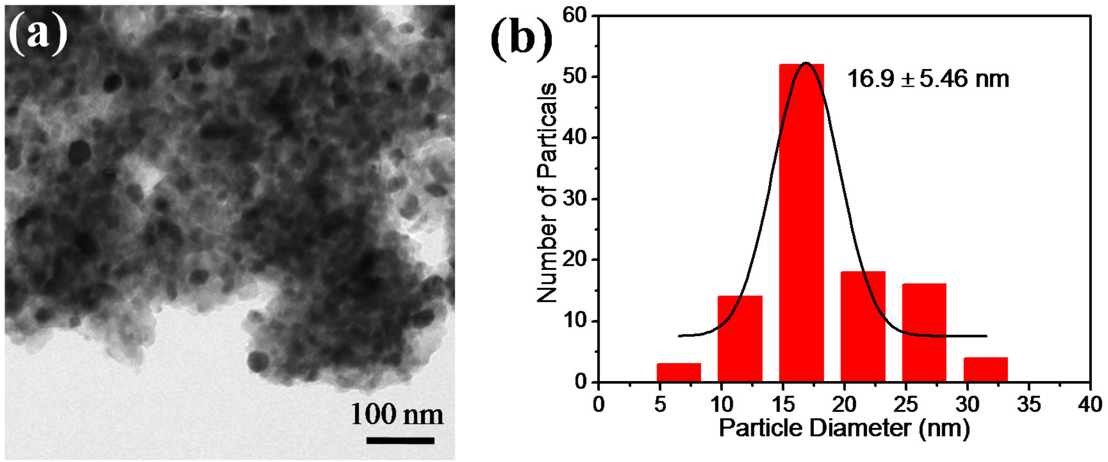

Fig. S2 (a) TEM image of small AuNPs/TiO_2 and (b) Diameter distribution of small sized AuNPs determined by measuring 100 individual AuNPs on TiO_2.

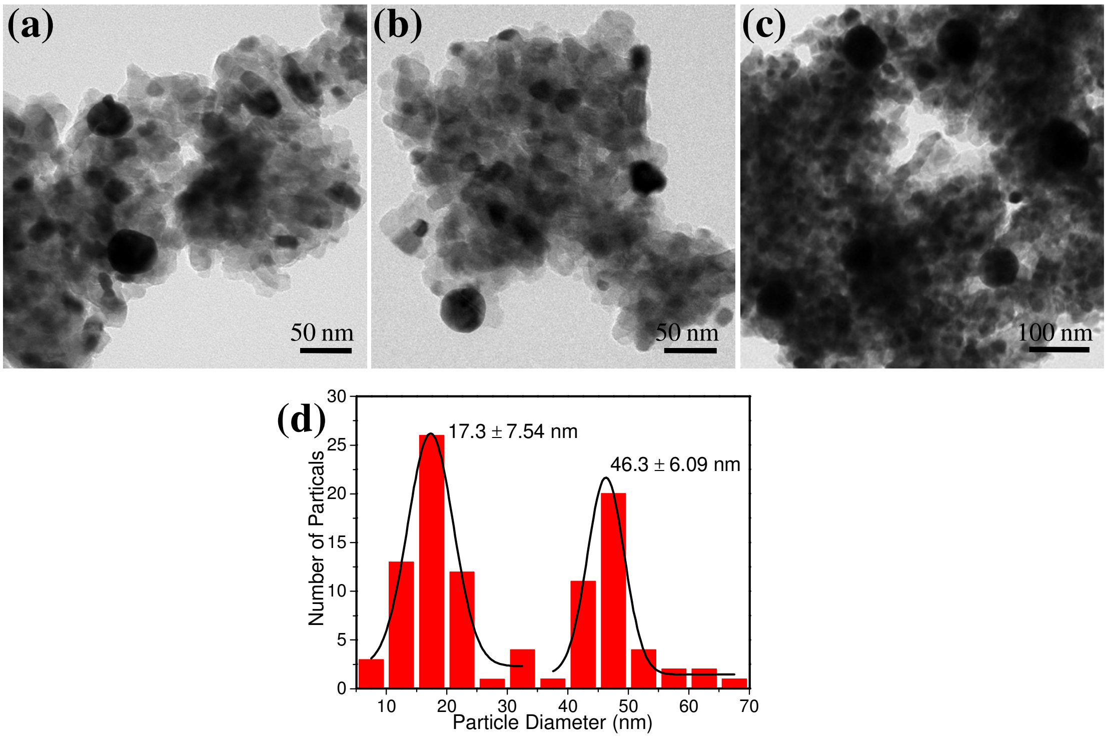

Fig. S3 (a-c) TEM images of dual particle-size AuNPs/TiO_2 and (d) Diameter distribution of dual particle-size AuNPs determined by measuring 100 individual AuNPs on TiO_2.

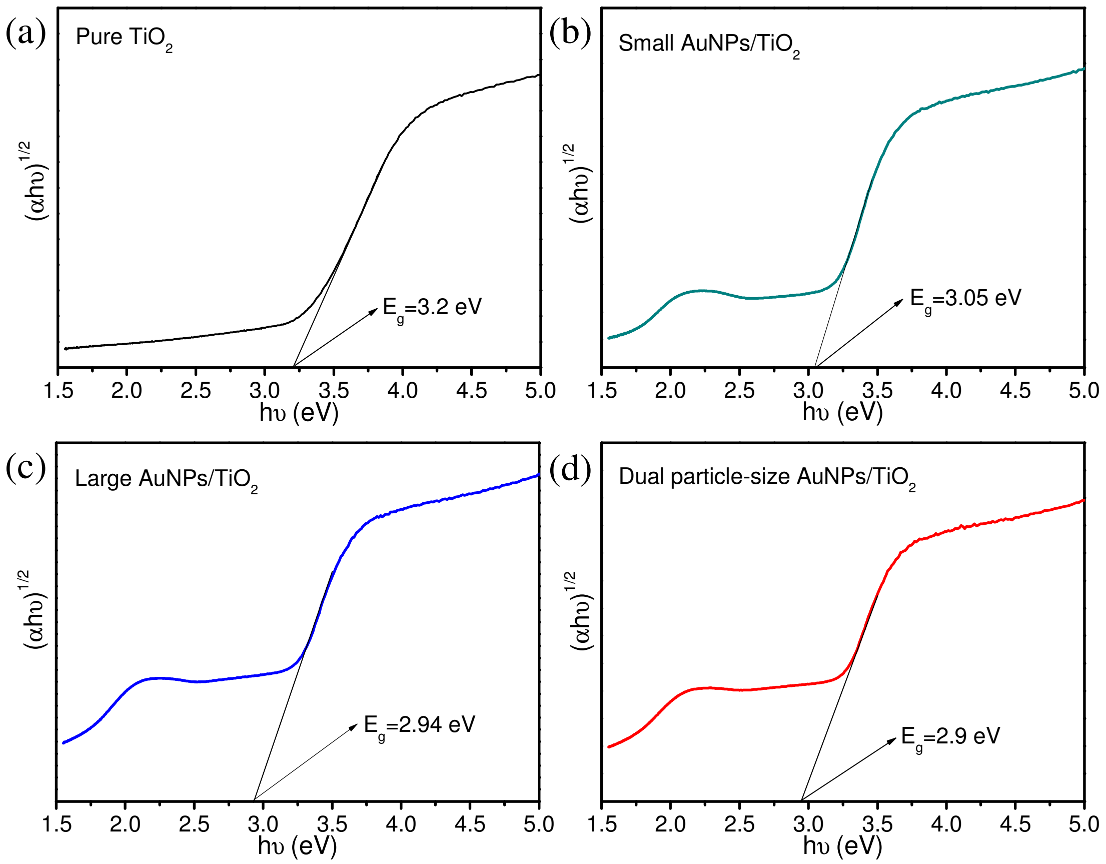

Fig. S4 Transformed Kubelka-Munk function vs. the light energy curves of (a) pure TiO_2, (b) small AuNPs/TiO_2, (c) large AuNPs/TiO_2 and (d) dual particle-size AuNPs/TiO_2.

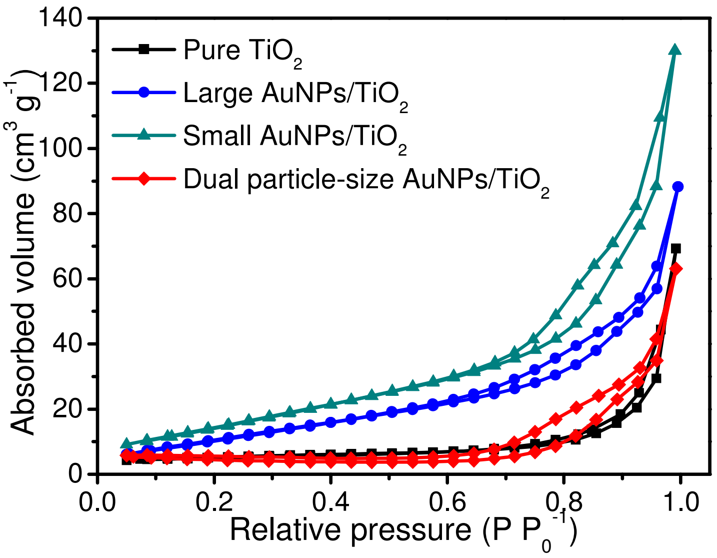

Fig. S5 Nitrogen adsorption of pure TiO_2, large AuNPs/TiO_2, small AuNPs/TiO_2 and dual particle-size AuNPs/TiO_2.

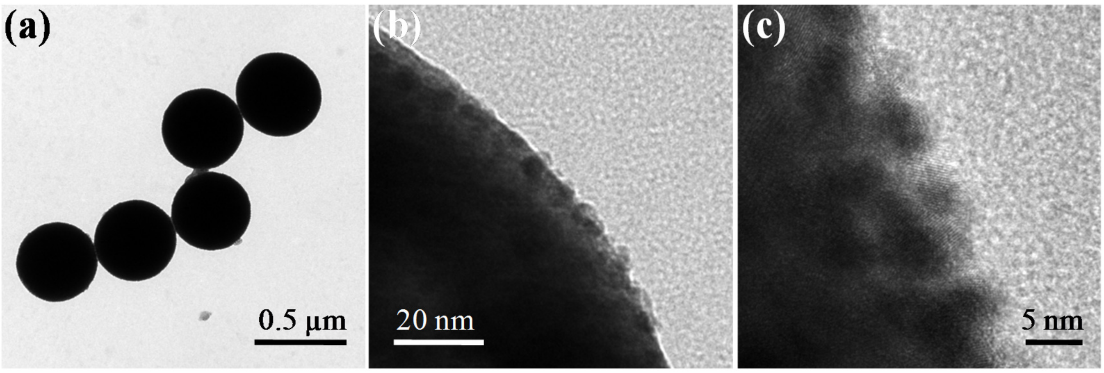

Fig. S6 (a) TEM and (b, c) HRTEM images of gold-dicyanodiamine composites (GDCs).

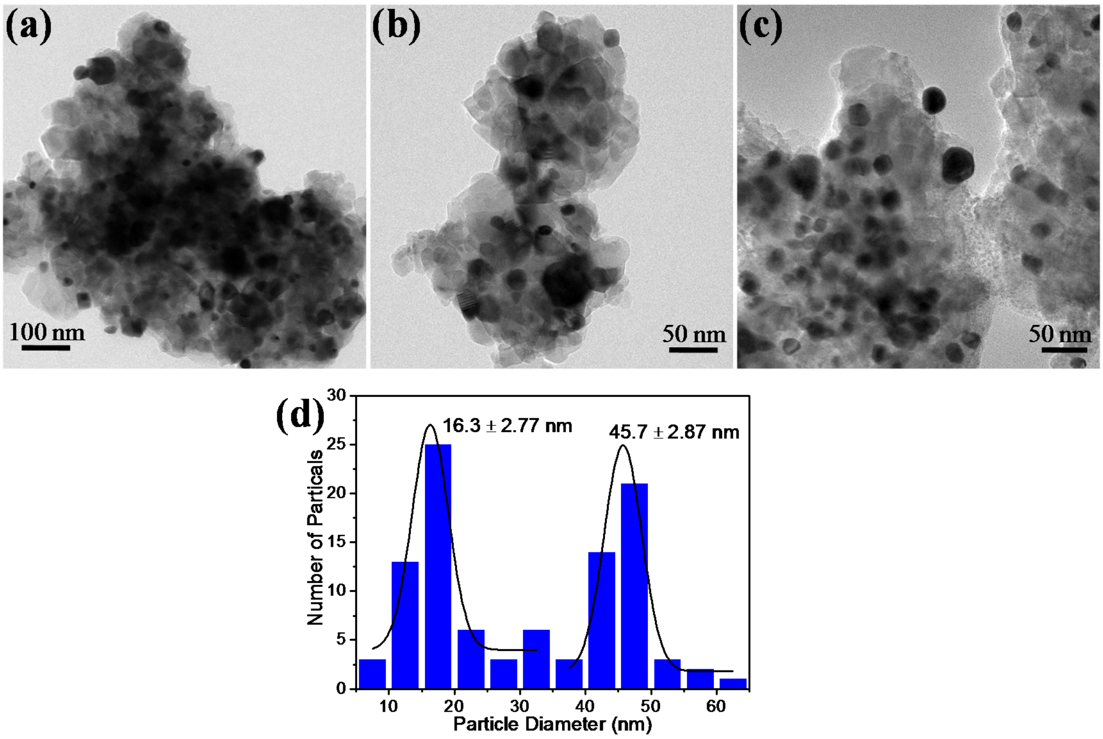

Fig. S7 (a-c) TEM images of 2.1 wt% GDC-directed dual particle-size AuNPs/TiO_2 and (d) Diameter distribution of 2.1 wt% GDC-directed dual particle-size AuNPs determined by measuring 100 individual AuNPs on TiO_2.

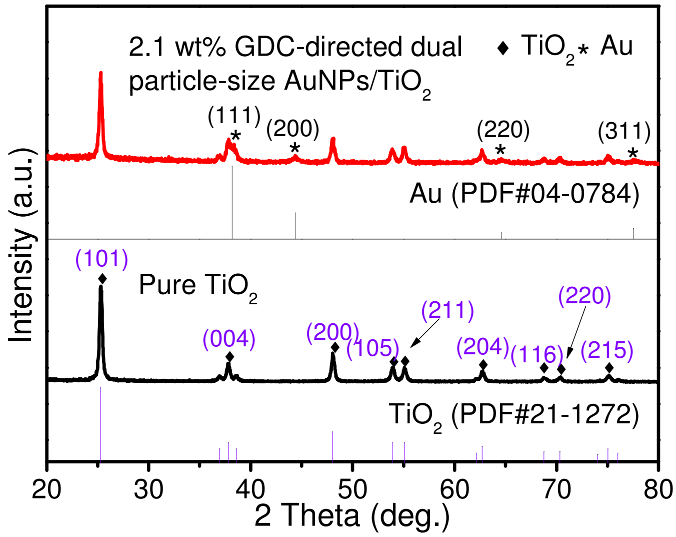

Fig. S8 XRD patterns of pure TiO_2 and 2.1 wt% GDC-directed dual particle-size AuNPs/TiO_2.

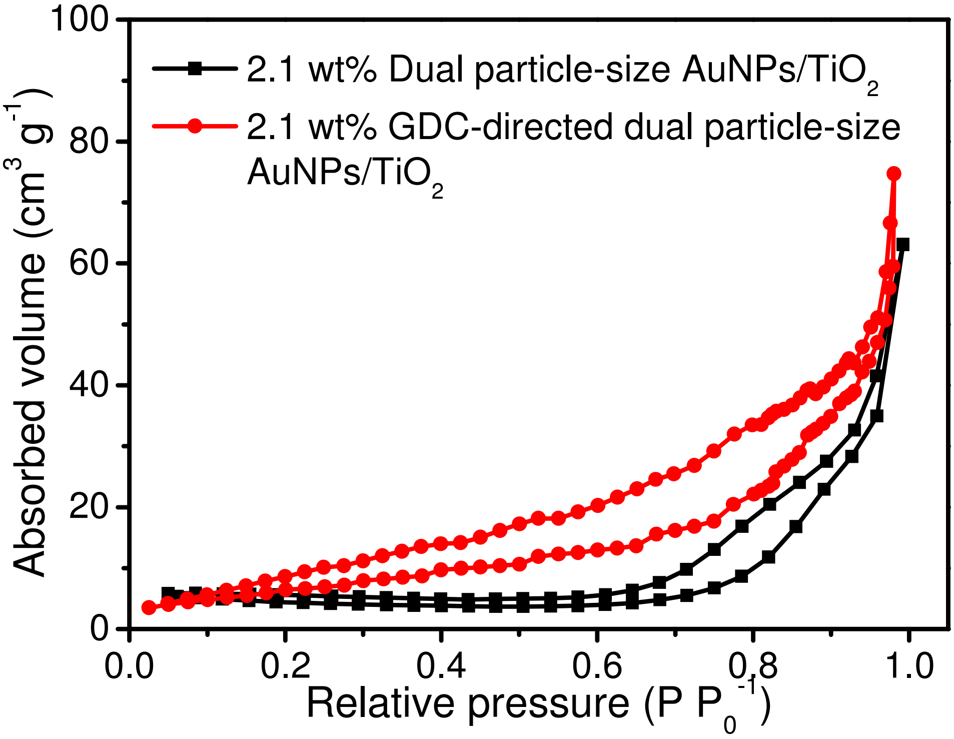

Fig. S9 Nitrogen adsorption of 2.1 wt% dual particle-size AuNPs/TiO_2 and 2.1 wt% GDC-directed dual particle-size AuNPs/TiO_2.

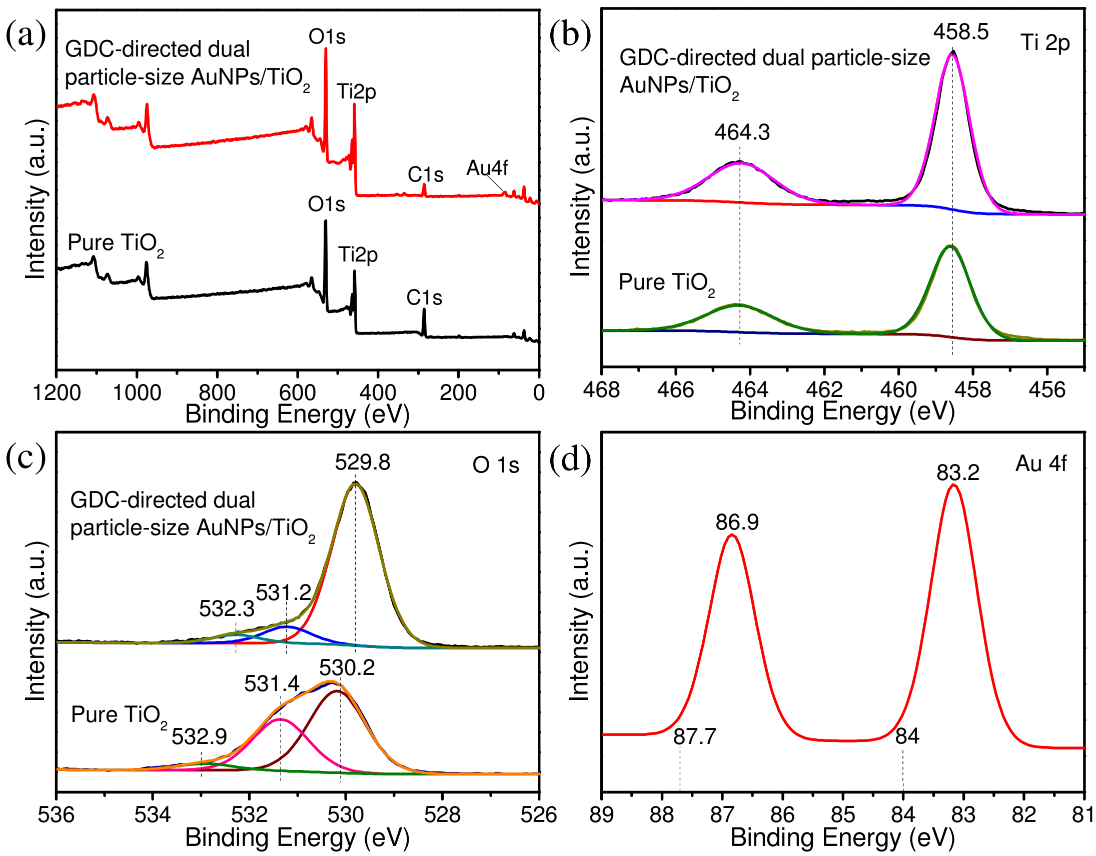

Fig. S10 (a) Survey XPS spectrum and high-resolution spectra of (b) Ti 2p, (c) O 1s and (d) Au 4f for pure TiO_2 and 2.1 wt% GDC-directed dual particle-size AuNPs/TiO_2.
